# Impact of Illicit Drug Use on Facial Fracture Patterns and Hospital Resource Utilization

**DOI:** 10.3390/cmtr18040051

**Published:** 2025-12-05

**Authors:** Arya Sherafat, Aishwarya Suresh, Ian Waldrop, Jared Inman

**Affiliations:** 1 School of Medicine, University of California, Riverside, CA 92507, USA; arya.sherafat@medsch.ucr.edu; 2 Department of Otolaryngology—Head and Neck Surgery, Loma Linda University Health, Loma Linda, CA 92354, USA; 3 Department of Surgery, Riverside Community Hospital, Riverside, CA 92501, USA

**Keywords:** facial fracture, trauma, drug intoxication

## Abstract

Introduction: Facial trauma is a public health concern, with reports of about 25% of all traumas having a component of facial injury. Alcohol and illicit drug use have previously been shown to exacerbate the severity of trauma injuries. This study investigates the relationship between illicit drug use and clinical outcomes amongst patients presenting with facial fracture injuries. Methods: A retrospective multicenter cohort study through the TriNetX database was performed. Drug Involved Facial fracture injury with illicit drug use (DIFFI+) was compared to a Non-Drug Involved Facial Fracture Injury group (DIFFI−). After propensity score matching, measures of hospital burden outcomes, surgical intervention, and underlying psychiatric diagnosis outcomes were compared. Results: A total of 27,863 propensity score-matched DIFFI+ cases were used for analysis. DIFFI+ patients were younger (mean age 33.8 vs. 42.0 years, *p* < 0.001), more often male (75% vs. 56%, *p* < 0.001), and more likely to be Black/African American (21% vs. 14%, *p* < 0.001), with cannabis and opioid use most common. DIFFI+ patients had a significantly higher odds of hospital burden outcomes, including psychiatry services (OR = 8.40), ventilator management (OR = 5.18), and critical care services (OR = 3.83). Conclusions/Discussion: DIFFI+ was significantly correlated with greater hospital burden in all analyzed clinical outcomes. DIFFI+ patients have a higher odds of receiving surgery but lower odds of receiving a fracture diagnosis. Having a psychiatric diagnosis increases risk for DIFFI+ injury.

## 1. Introduction

Traumatic injuries have been shown to be among the costliest health problems in the United States [1]. It is estimated that 25% of all trauma involves a component of facial injury [2,3]. Thus, facial trauma can have significant public health implications. Substance use, including alcohol and illicit drugs, has been associated with trauma-related injuries. It is reported that 35–80% of all trauma patients tested positive for an illicit drug [4]. Illicit drug use can have many consequences on a patient’s overall health, recovery, and in-hospital care. For instance, illicit drug use, especially if undisclosed, can increase the risk of drug interactions and side effects of medications and general anesthesia [4]. Moreover, it has been established that patients who were drug use positive had more facial fractures and lacerations as well as longer hospital stays and more allied health inputs than drug use negative patients with facial trauma [5]. A National Electronic Injury Surveillance System database study by Brozynski et al. found that patients who presented to the emergency department with plastic surgery-related injuries, including facial injuries, had higher rates of hospital admission when psychoactive drugs had been used [6]. These previous studies indicate that patients with facial trauma and illicit drug use may have a larger burden on the healthcare system due to increased care needs and use of resources. It has also been reported that despite positive illicit drug screens or admitted illicit drug use amongst trauma patients, many patients are not referred to early intervention or other prevention programs, highlighting the risk of persistent use and reinjury [1,7]. Though there are some smaller studies that exist in the literature examining the relationship of illicit drug use and facial trauma and its implications amongst specific populations in the United States, there is no large database study on this topic that is representative of the general population of the United States [7,8,9]. As trauma patterns and illicit drug use can vary throughout different geographic locations with varying socioeconomic environments, it is important to draw upon a database with diverse populations to thoroughly understand the relationship of illicit drug use and facial trauma with its impacts on the healthcare system and society. This information can help identify factors to aid in building effective interventions and prevention strategies that may currently be underutilized or unfamiliar to many clinicians. This study aims to examine the impact of illicit drug use on facial fracture patterns and compare demographic factors, hospital burden outcomes, fracture type, and underlying psychiatric diagnosis amongst facial fractures using a large, multi-institutional database.

## 2. Materials and Methods

### 2.1. Data Source

This study utilizes TriNetX, a global federated research database with de-identified patient electronic medical record data from more than 120 healthcare organization (HCO) collaborators. Using the United States (US) collaborative network, representative of about 70 HCOs within the US, a retrospective multicenter cohort study was conducted. This study uses all available data in the TriNetX database, and queries were run in May 2025. The database was queried for all patients who presented to an HCO with a primary outcome of a facial fracture. Inclusion for this group included patients of any age who presented with a code for fracture of mandible; fracture of orbital floor; fracture of nasal bones; fracture of malar, maxillary, and zygoma bones with an additional diagnosis of drug use, including cannabis, opioid, cocaine, stimulant, inhalant, sedative, or hallucinogen within 24 h of presentation. These groups were classified as Drug Involved Facial Fracture Injury (DIFFI+). The study compared facial fractures with each individual drug (DIFFI+Drug) to a group of facial fractures without any drug use within 24 h (DIFFI−). The DIFFI− group excluded all instances of illicit drug use (cannabis, opioid, cocaine, stimulant, inhalant, sedative, or hallucinogen). Patients with multiple diagnoses of facial fractures were aggregated and represented as a single case within the cohort. Queries were run in accordance with the common procedural terminology (CPT) and the international classification of diseases (ICD) to minimize variability in data capture between participating HCOs (Supplemental Table S1).

### 2.2. Study Design and Propensity Matching

Cohorts were propensity score matched by age at injury, sex, and race (White, Black/African American, Hispanic/Latino). Binary variables were balanced by presence or absence, and age was balanced as a continuous variable. Cohorts were propensity-matched 1:1 according to a validated matching algorithm, which yielded evenly divided cohorts for each drug group (Supplemental Table S2).

### 2.3. Statistical Analysis

The primary outcomes of this study were categorized by hospital burden measures, injury classification/categorization, procedure following injury, and underlying psychiatric diagnosis. Statistical analysis using Microsoft Excel (Version 16.101.2) was performed to calculate risk ratios, odds ratios, and CIs comparing 18 different outcomes (Supplemental Table S3). To evaluate hospital burden outcomes, odds ratios were used from each drug category outcome (DIFFI+Drug) and averaged to represent the DIFFI+ population. Differences between drug types were further evaluated. Demographic factor differences were evaluated, analyzed, and compared prior to propensity score matching. After propensity score matching, odds ratios with 95% CIs were used to draw conclusions regarding hospital burden outcomes, fracture classification, and procedures. Risk Ratio was used to evaluate the underlying psychiatric diagnosis of those who presented with a DIFFI+Drug. Outcomes with a *p* < 0.05 were deemed statistically significant.

## 3. Results

### 3.1. DIFFI+ Demographics

When evaluating DIFFI+, before matching, DIFFI+ patients were younger at the time of injury, with a mean age of 39.8, compared to the DIFFI− group at 42.0 (*p* < 0.001). Male patients were more likely to experience DIFFI+ (75% vs. 56%, *p* < 0.001) and comprise the majority of DIFFI+ injury. Female patients were less likely to experience DIFFI+ (21% vs. 37%, *p* < 0.001). DIFFI+ was compared to the DIFFI− group to find that White patients were less likely to experience DIFFI+ (59% vs. 65%, *p* < 0.001), Black or African American patients were more likely to experience DIFFI+ (21% vs. 14%, *p* < 0.001), with no significant differences in Hispanic/Latino patients.

**Figure 1:**
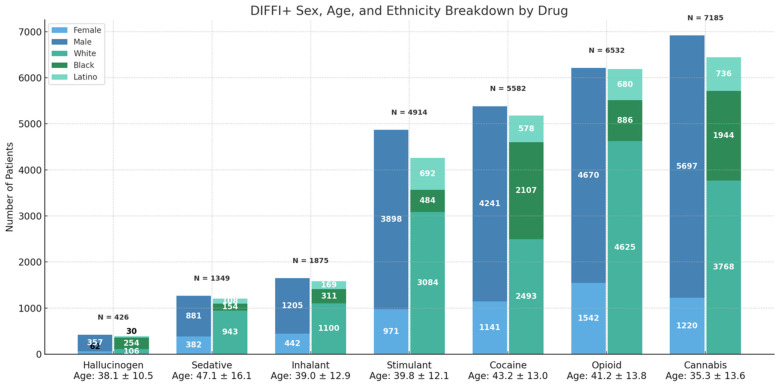
Sex, age, and ethnicity distributions of patients with facial fracture injury stratified by associated drug exposure. Bars represent the total number of patients in each drug category and further subdivided by demographic characteristics. Mean age at time of injury is shown below each drug category.

When identifying demographics of DIFFI+ amongst specific drugs, it is evident that DIFFI+Cannabis occurs most frequently (n = 7185), followed by DIFFI+Opioid (n = 6532), DIFFI+Cocaine (n = 5582), DIFFI+Stimulant (n = 4914), DIFFI+Inhalant (n = 1875), DIFFI+Sedative (n = 1349), and lastly DIFFI+Hallucinogen (n = 426). DIFFI+Sedatives have the oldest average age (47.1 ± 16.1), while DIFFI+Cannabis has the youngest average age (35.3 +/− 13.6) (Figure 1). DIFFI+Cocaine injury occurred at significantly higher rates in Black/African American populations compared to DIFFI− in the same race category (37.7% vs. 14.4%, *p* < 0.001). White patients comprise the greater proportion of DIFFI+Opioid (n = 4625, 70.8%), closely followed by DIFFI+Sedative (n = 943, 69.9%).

### 3.2. DIFFI and Hospital Burden

**Figure 2:**
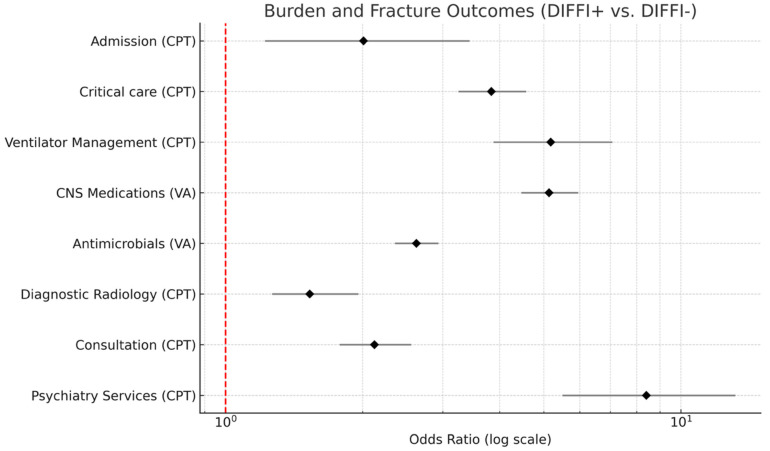
Forest plot displaying odds ratio (log scale) for hospital burden and fracture related outcomes among DIFFI+ vs. DIFFI− patients. Diamonds represent an estimated odds ratio amongst all drug groups, and horizontal lines denote a 95% confidence interval. The red dashed line indicates the null value (OR = 1).

DIFFI+ showed a significantly increased odds of hospital burden outcomes in all categories (Figure 2). All values in the forest plot (Figure 2) greater than 1 (red dashed line) represent the increased odds outcomes in the DIFFI+ group.

DIFFI+ patients had the highest odds of receiving psychiatry services (OR = 8.40; 95% CI: 5.49–13.17). This group also experienced increased odds of ventilator management (OR = 5.18; 95% CI: 3.88–7.07), central nervous system (CNS) medications (OR = 5.13; 95% CI: 4.46–5.95), and critical care/intensive care services (OR = 3.83, 95% CI: 3.24–4.57) at similar rates. Additionally, antimicrobials (OR = 2.63; 95% CI: 2.36–2.93), consultation (OR = 2.12; 95% CI: 1.78–2.56), admission (OR = 2.01, 95% CI: 1.22–3.43), and diagnostic radiology (OR = 1.53; 95% CI: 1.26–1.96) all had higher odds (Supplemental Table S4).

When looking at drug specific differences in DIFFI+ patients, it is evident that DIFFI+Sedatives have higher odds compared to average of all drug groups specifically in ventilation management (OR = 8.85, 95% CI: 5.68–13.70), consults (OR = 3.31, 95% CI: 2.59–4.24), and psychiatry services (OR = 13.33, 95% CI: 6.94–25.64). DIFFI+Hallucinogens have lower odds compared to the average of all drug groups in antimicrobials (OR = 1.53, 95% CI: 1.17–2.00), psychiatry consults (OR = 3.27; 95% CI: 1.58–6.76), and critical care/intensive care services (OR = 2.12, 95% CI: 1.40–3.21). DIFFI+Stimulants, although still greater odds than DIFFI−, have a lower odds of being admitted compared to other drugs (OR = 1.39; 95% CI: 0.91–2.14).

### 3.3. DIFFI+ Surgical Procedures and Diagnosis

**Figure 3:**
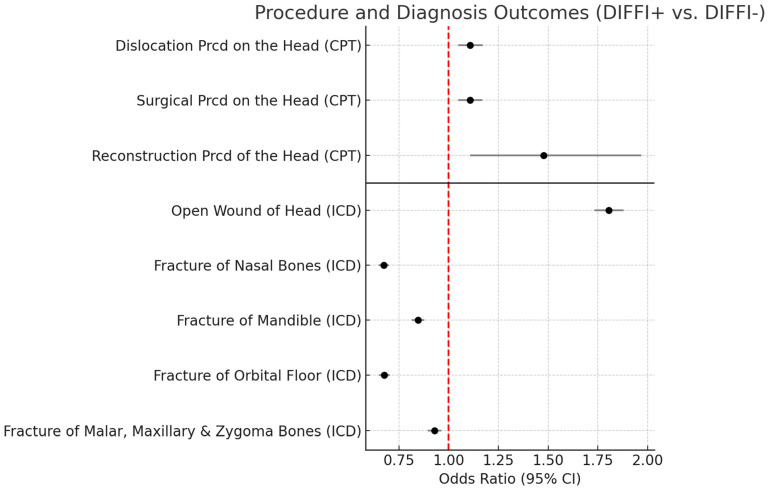
Forest plot illustrating procedure and diagnosis outcomes between DIFFI+ vs. DIFFI− patients. Points represent estimated odds ratios, and horizontal lines indicate 95% confidence intervals. The red dashed vertical line denotes the null value (OR = 1).

DIFFI+ experienced greater odds of fracture and/or dislocation procedures (open/closed reduction) on the head (OR = 1.11; 95% CI: 1.05–1.17), surgical procedures on the head (OR = 1.11; 95% CI: 1.05–1.17), and reconstruction procedures on the head (OR = 1.48; 95% CI: 1.11–1.97). DIFFI+ experienced a higher diagnosis of open wound of the head (OR = 1.81; 95% CI: 1.73–1.88). Alternatively, DIFFI+ experienced reduced odds of having a diagnosis outcome of fracture of nasal bones (OR = 0.67; 95% CI: 0.65–0.67), fracture of mandible (OR = 0.85; 95% CI: 0.81–0.88), fracture of orbital floor (OR = 0.68; 95% CI: 0.65–0.70), or fracture of malar, maxillary and zygoma bones (OR = 0.93; 95% CI: 0.90–0.96) (Figure 3). All values in the forest plot (Figure 3) greater than 1 (red dashed line) represent the increased odds outcomes in the DIFFI+ group. Values less than 1 represent decreased odds outcomes in the DIFFI+ group.

### 3.4. DIFFI and Psychiatric Diagnosis Risk

**Figure 4:**
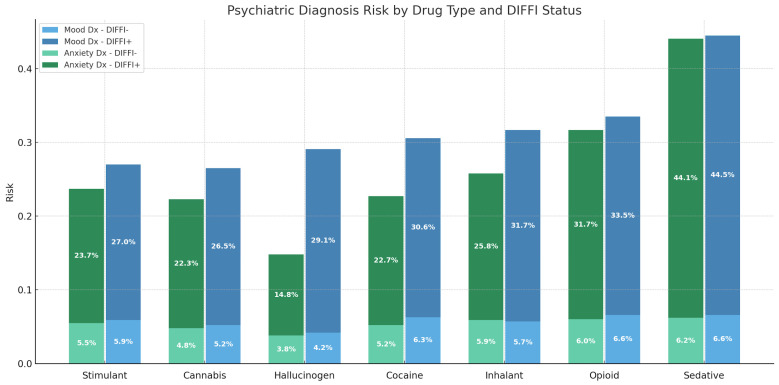
Psychiatric diagnosis risk by drug type and DIFFI status. Bar plot showing the risk of patients with a mood and anxiety disorder among DIFFI+ and DIFFI− cohorts across all drug categories. DIFFI+ patients consistently demonstrated higher risk of both mood and anxiety diagnosis across all substance types.

DIFFI+Sedatives are associated with the greatest increased risk of having an underlying mood (RD = −37.88%, 95% CI: −40.85–−34.92, *p* < 0.001) or anxiety (RD = −37.83%, 95% CI: −40.85–−34.94, *p* < 0.001) disorder. All drug groups displayed a statistically significant (*p* < 0.05) risk difference between DIFFI+Drug vs. DIFFI− when comparing mood and anxiety disorder outcomes (Figure 4).

## 4. Discussion

This study provides an analysis of a nationally representative population of patients with DIFFI+ and reveals several important trends in demographic features, public health resource utilization, and association with mood and anxiety disorders. It was found that a significantly higher number of males are affected by DIFFI+ than females. This is consistent with what has been previously reported in the literature regarding alcohol related injuries as well as prior retrospective studies examining substance use and facial trauma [10,11]. There were several significant differences in drugs associated with DIFFI+ amongst various demographic groups, but of all substances, cannabis was the most frequently used amongst patients with DIFFI+. As policies on the legalization of cannabis continue to evolve, this may have important implications for the rate and severity of facial trauma. It has been shown that when comparing facial trauma before and after the legalization of marijuana in Colorado, there was an increase in rates of maxillary and skull base fractures [12]. In general, patients with DIFFI+ were found to have a larger hospital burden, including more frequent admissions and greater use of multidisciplinary consultations, critical care management, antimicrobials, and radiology services. The significantly increased need for in-hospital resources and admissions amongst patients with DIFFI+ shown by this data highlights the need for prevention-based strategies not only from a patient safety standpoint but also from the perspectives of healthcare utilization and cost effectiveness. Amongst the various substances associated with injury, sedatives were associated with the greatest need for ventilation, specialty consultation, and psychiatric services, while patients using stimulants were the least likely to be admitted following DIFFI+. DIFFI+ patients with sedative use had the greatest risk of having underlying mood or anxiety disorders. This study also found that sedatives were more frequently used by white patients and that DIFFI+ associated with cocaine occurred more frequently in African American or black patients. These findings suggest that sedative use may be associated with more severe trauma or be associated with injuries yielding multiple comorbid conditions. Alternatively, given the associations with sedative use and white patients with DIFFI+ as compared to cocaine use and African American patients with DIFFI+, it is important to consider the role racial disparities and differences play in subsequent admission and provision of healthcare resources. A national database study has previously found that, amongst patients presenting to the emergency department, black patients were 10% less likely to be admitted to the hospital when compared to white patients [13]. Thus, accounting for race-based disparities is essential when understanding the allocation of healthcare resources amongst patients with DIFFI+.

When examining the need for surgical procedures, the data showed that the DIFFI+ patients when compared to DIFFI− have significantly more surgical procedures. However, patients with DIFFI+ were also less frequently diagnosed with a facial fracture. This suggests the DIFFI− population may be self-evaluating and coming to the emergency department less frequently for minor facial trauma, whereas patients with DIFFI+ may be more likely to present to the emergency department for any amount of injury, even if considered minor and unlikely to have significant trauma or fractures. Further, patients with DIFFI+ may be coming into the hospital for other secondary reasons related to their drug use or comorbidities rather than due to concern for facial trauma.

The presence of an underlying mood or anxiety disorder increased the risk of experiencing a DIFFI+, and this was true for all drug groups, with sedatives having the highest risk. A multidisciplinary approach to prevention is needed to address these underlying demographic factors, psychiatric comorbidities, and patterns of drug use that underlie DIFFI+.

Given the significant hospital burden and psychiatric comorbidities identified amongst patients with DIFFI+, implementation of early preventative strategies becomes important for clinical applications. Previous studies have established the emergency department as an opportunity to capitalize on illicit drug use screening for high-risk individuals [14]. This study supports these screening protocols and encourages early mental health or substance use referrals for patients who are at higher risk for facial trauma. Community-based injury prevention programs might further mitigate the incidence of illicit drug-related facial fractures.

There are limitations to this study that are important to note. TriNetX is an administrative database, and therefore, the data input by participating institutions might vary between providers. This might lead to coding inconsistencies, which might ultimately alter the data quality. This was addressed by selecting diagnostic and procedural codes using standardized ICD-10 and CPT to minimize variability between HCOs (Supplemental Table S1). Additionally, diagnostic coding limitations create a lack of granular clinical details and limited longitudinal follow-up data. Future directions of this research can include evaluating specific long-term outcomes in DIFFI+ patient populations.

Overall, this study provides a framework for understanding the burden of DIFFI+ on the healthcare system based on a nationally representative database, offering valuable data that can shape healthcare policy, resource allocation, and preventative initiatives.

## Data Availability

Data can be made available through email of corresponding author.

## Supplementary Materials

The following supporting information can be downloaded at: https://www.mdpi.com/article/10.3390/cmtr18040051/s1. Table S1: ICD-10-CM diagnosis codes used in analysis; Table S2: Pre-matched and post-matched demographic data; Table S3: ICD-10-CM, CPT, and VA Outcome codes used in analysis; Table S4: Specific outcome results by drug type.
