# Supplementary material for: Impact of Illicit Drug Use on Facial Fracture Patterns and Hospital Resource Utilization
Source: Craniomaxillofac Trauma Reconstr. 2025 Dec 5;18(4):51. doi: 10.3390/cmtr18040051 (PMC12732292; doi:10.3390/cmtr18040051)
Supplement: Supplementary file 1 [file cmtr-18-00051-s001.zip › cmtr-3957733-supplementary.pdf]

Supplementary Materials:

Supplemental Table S1. Diagnosis Codes	
**Diagnosis**	**ICD-10-CM Code**
Fracture of mandible	S02.6
Fracture of malar, maxillary and zygoma bones	S02.4
Fracture of orbital floor	S02.3
Fracture of nasal bones	S02.2
Opioid abuse, uncomplicated	F11.10
Opioid dependence, uncomplicated	F11.20
Cannabis abuse, uncomplicated	F12.10
Cannabis dependence, uncomplicated	F12.20
Other stimulant abuse, uncomplicated	F15.10
Other stimulant dependence, uncomplicated	F15.20
Cocaine abuse, uncomplicated	F14.10
Cocaine dependence, uncomplicated	F14.20
Hallucinogen abuse, uncomplicated	F16.10
Hallucinogen dependence, uncomplicated	F16.20
Sedative, hypnotic or anxiolytic dependence, uncom	F13.20
Sedative, hypnotic or anxiolytic abuse, uncomplicat	F13.10
Inhalant abuse, uncomplicated	F18.10
Inhalant dependence, uncomplicated	F18.20

Supplemental Tabel S2a. Pre-Matched Demographic Data

Drug	N (DIFI-)	N (DIFI+)	Mean Age (DIFI	Mean Age (DIFI	Male (DIFI-)	Male (DIFI+)	Female (DIFI-)	Female (DIFI+)	White (DIFI-)	White (DIFI+)	Black (DIFI-)	Black (DIFI+)	Latino (DIFI-)	Latino (DIFI+)
Cannabis	826212	7185	41.6 +/- 23.5	35.3 +/- 13.6	481952	5697	302420	1220	529426	3768	118708	1944	88369	736
Cocaine	826212	5582	41.6 +/- 23.5	43.2 +/- 13.0	481952	4241	302420	1141	529426	2493	118708	2107	88369	578
Hallucinoger	826212	426	41.6 +/- 23.5	38.1 +/- 10.5	481952	357	302420	62	529426	106	118708	254	88369	30
Inhalant	826212	1875	41.6 +/- 23.5	39.0 +/- 12.9	481952	1205	302420	442	529426	1100	118708	311	88369	169
Opioid	826212	6532	42.6 +/- 23.5	41.2 +/- 13.8	481952	4670	302420	1542	529426	4625	118708	886	88369	680
Sedative	826212	1349	49.0 +/- 22.8	47.1 +/- 16.1	481952	881	302420	382	529426	943	118708	154	88369	108
Stimulant	826212	4914	41.6 +/- 23.5	39.8 +/- 12.1	481952	3898	302420	971	529426	3084	118708	484	88369	692

Supplemental Table S2b. Post-Matched Data Data

Drug	N (DIFI-)	N (DIFI+)	Mean Age (DIFI	Mean Age (DIFI	Male (DIFI-)	Male (DIFI+)	Female (DIFI-)	Female (DIFI+)	White (DIFI-)	White (DIFI+)	Black (DIFI-)	Black (DIFI+)	Latino (DIFI-)	Latino (DIFI+)
Cannabis	7185	7185	35.3 +/- 13.6	35.3 +/- 13.6	5698	5697	1220	1220	3768	3768	1944	1944	736	736
Cocaine	5582	5582	43.2 +/- 13.0	43.2 +/- 13.0	4242	4241	1141	1141	2494	2493	2107	2107	578	578
Hallucinoger	426	426	38.1 +/- 10.5	38.1 +/- 10.5	357	357	62	62	106	106	254	254	30	30
Inhalant	1875	1875	39.0 +/- 12.9	39.0 +/- 12.9	1205	1205	442	442	1100	1100	311	311	168	168
Opioid	6532	6532	41.2 +/- 13.8	41.2 +/- 13.8	4670	4670	1542	1542	4625	4625	886	886	680	680
Sedative	1349	1349	47.1 +/- 16.1	47.1 +/- 16.1	881	881	382	382	943	943	158	158	108	108
Stimulant	4914	4914	39.8 +/- 12.1	39.8 +/- 12.1	3898	3898	971	971	3084	3084	484	484	692	692

Supplemental Table S3. Outcome Codes		
**Diagnosis**	**Code Type**	**Code**
Mood [affective] disorders	ICD-10-CM	F30-F39
Anxiety, dissociative, stress-related, somatoform and other nonps	ICD-10-CM	F40-F48
Fracture of orbital floor	ICD-10-CM	S02.3
Fracture of nasal bones	ICD-10-CM	S02.2
Fracture of malar, maxillary and zygoma bones	ICD-10-CM	S02.4
Fracture of mandible	ICD-10-CM	S02.6
Open wound of head	ICD-10-CM	S01
**Procedure**	**Code Type**	**Code**
Repair, Revision, and/or Reconstruction Procedures on	CPT	1003833
Fracture and/or Dislocation Procedures on the Head	CPT	1003918
Surgical Procedures on the Head	CPT	1003799
Consultations	CPT	1013686
Ventilator Management	CPT	1015098
Critical Care Services	CPT	1013729
Hospital Inpatient or Observation Care Services (Including Admis	CPT	1013675
Diagnostic Radiology (Diagnostic Imaging) Procedures	CPT	1013252
Psychiatry Services and Procedures	CPT	1012681
**Medication**	**Code Type**	**Code**
Central nervous system medications	VA	CN000
Antimicrobials	VA	AM000

Supplemental Table S4a. Odds by Drug

Admission

Substance	Odds Ratio	CI Lower	CI Upper
Stimulant	1.39275766	0.9057971	2.14132762
Opioid	2.75482094	1.91570881	3.96825397
Sedative	2.42718447	1.15473441	5.10204082
Inhalant	1.92678227	0.9569378	3.89105058
Hallucinogen	1	0.41186161	2.42718447
Cocaine	2.1978022	1.5015015	3.21543408
Cannabis	2.36406619	1.69491525	3.30033003

Critical Care

Substance	Odds Ratio	CI Lower	CI Upper
Stimulant	4.85436893	4.31034483	5.46448087
Opioid	4.18410042	3.7593985	4.65116279
Sedative	5.46448087	4.32900433	6.94444444
Inhalant	3.06748466	2.49376559	3.77358491
Hallucinogen	2.11864407	1.40252454	3.20512821
Cocaine	3.78787879	3.37837838	4.23728814
Cannabis	3.36700337	3.03951368	3.73134328

Ventilator Mngmnt

Substance	Odds Ratio	CI Lower	CI Upper
Stimulant	7.04225352	5.74712644	8.62068966
Opioid	5.20833333	4.32900433	6.28930818
Sedative	8.84955752	5.68181818	13.6986301
Inhalant	2.52525253	1.7211704	3.7037037
Hallucinogen	2.51889169	1.26742712	5
Cocaine	5.07614213	4.16666667	6.21118012
Cannabis	5.02512563	4.21940928	5.98802395

CNS medications

Substance	Odds Ratio	CI Lower	CI Upper
Stimulant	7.40740741	6.57894737	8.33333333
Opioid	6.36942675	5.81395349	7.04225352
Sedative	6.84931507	5.55555556	8.47457627
Inhalant	2.80898876	2.43309002	3.24675325
Hallucinogen	3.02114804	2.18340611	4.18410042
Cocaine	4.40528634	4	4.85436893
Cannabis	5.07614213	4.65116279	5.52486188

Antimicrobials

Substance	Odds Ratio	CI Lower	CI Upper
Stimulant	3.84615385	3.5335689	4.18410042
Opioid	2.96735905	2.76243094	3.18471338
Sedative	2.80112045	2.3923445	3.26797386
Inhalant	1.85528757	1.6286645	2.1141649
Hallucinogen	1.52905199	1.16686114	2.00400802
Cocaine	2.54452926	2.35849057	2.74725275
Cannabis	2.83286119	2.64550265	3.03030303

Diagnostic Radiology

Substance	Odds Ratio	CI Lower	CI Upper
Stimulant	2.31481481	2.10526316	2.54452926
Opioid	1.37931034	1.28040973	1.4858841
Sedative	1.47710487	1.25313283	1.74216028
Inhalant	1.92678227	0.9569378	3.89105058
Hallucinogen	0.53276505	0.40096231	0.7082153
Cocaine	1.49925037	1.38121547	1.62601626
Cannabis	1.58478605	1.47492625	1.70357751

Consultation

Substance	Odds Ratio	CI Lower	CI Upper
Stimulant	2.04498978	1.8018018	2.32018561
Opioid	2.3364486	2.09205021	2.61096606
Sedative	3.31125828	2.59067358	4.23728814
Inhalant	0.96432015	0.78926598	1.1778563
Hallucinogen	1.31926121	0.82987552	2.09643606
Cocaine	2.51889169	2.23713647	2.83286119
Cannabis	2.36406619	2.12314225	2.62467192

Psychiatry Services

Substance	Odds Ratio	CI Lower	CI Upper
Stimulant	9.17431193	6.4516129	12.987013
Opioid	9.17431193	6.84931507	12.195122
Sedative	13.3333333	6.94444444	25.6410256
Inhalant	5.84795322	3.47222222	9.80392157
Hallucinogen	3.26797386	1.57977883	6.75675676
Cocaine	7.46268657	5.40540541	10.3092784
Cannabis	10.5263158	7.75193798	14.4927536

Supplemental Table S4b. Averaged Odds

Outcome	Odds Ratio	CI Lower	CI Upper
Admission	2.01	1.22	3.44
Critical Care	3.83	3.24	4.57
Ventilator Mngmnt	5.18	3.88	7.07
CNS medications	5.13	4.46	5.95
Antimicrobials	2.63	2.36	2.93
Diagnostic Radiology	1.53	1.26	1.96
Consultation	2.12	1.78	2.56
Psychiatry Services	8.40	5.49	13.17
